# Sustainability of a community-based anti-retroviral care delivery model – a qualitative research study in Tete, Mozambique

**DOI:** 10.7448/IAS.17.1.18910

**Published:** 2014-10-06

**Authors:** Freya Rasschaert, Tom Decroo, Daniel Remartinez, Barbara Telfer, Faustino Lessitala, Marc Biot, Baltazar Candrinho, Wim Van Damme

**Affiliations:** 1 Department of Public Health, Institute of Tropical Medicine, Antwerp, Belgium; 2 Médecins Sans Frontières, Tete, Mozambique; 3 Médecins Sans Frontières, Maputo, Mozambique; 4 Médecins Sans Frontières, Brussels, Belgium; 5 Ministry of Health, Tete, Mozambique; 6 School of Public Health, University of the Western Cape, Cape Town, South Africa

**Keywords:** community participation, ART, HIV, sustainability, patient empowerment, Mozambique

## Abstract

**Introduction:**

To overcome patients’ reported barriers to accessing anti-retroviral therapy (ART), a community-based delivery model was piloted in Tete, Mozambique. Community ART Groups (CAGs) of maximum six patients stable on ART offered cost- and time-saving benefits and mutual psychosocial support, which resulted in better adherence and retention outcomes. To date, Médecins Sans Frontières has coordinated and supported these community-driven activities.

**Methods:**

To better understand the sustainability of the CAG model, we developed a conceptual framework on sustainability of community-based programmes. This was used to explore the data retrieved from 16 focus group discussions and 24 in-depth interviews with different stakeholder groups involved in the CAG model and to identify factors influencing the sustainability of the CAG model.

**Results:**

We report the findings according to the framework's five components. (1) The CAG model was designed to overcome patients’ barriers to ART and was built on a concept of self-management and patient empowerment to reach effective results. (2) Despite the progressive Ministry of Health (MoH) involvement, the daily management of the model is still strongly dependent on external resources, especially the need for a regulatory cadre to form and monitor the groups. These additional resources are in contrast to the limited MoH resources available. (3) The model is strongly embedded in the community, with patients taking a more active role in their own healthcare and that of their peers. They are considered as partners in healthcare, which implies a new healthcare approach. (4) There is a growing enabling environment with political will and general acceptance to support the CAG model. (5) However, contextual factors, such as poverty, illiteracy and the weak health system, influence the community-based model and need to be addressed.

**Conclusions:**

The community embeddedness of the model, together with patient empowerment, high acceptability and progressive MoH involvement strongly favour the future sustainability of the CAG model. The high dependency on external resources for the model's daily management, however, can potentially jeopardize its sustainability. Further reflections are required on possible solutions to solve these challenges, especially in terms of human resources.

## Introduction

Since 2003, Médecins Sans Frontières (MSF) has supported the anti-retroviral therapy (ART) programme of the Ministry of Health (MoH) in Tete province, Mozambique. Despite decentralizing the HIV activities to the peripheral health facilities to improve access to ART, more than 20% of the patients remain lost to follow-up (LFU) and the majority of the people living with HIV (PLHIV) in remote areas are not able to access ART [1].

In many parts of the country, the major challenges to access ART and retain people on treatment are large distances and high transport costs, long waiting times in health facilities, poor relationships between patients and the health staff, and cultural beliefs such as traditional medicine [2–4].

In 2008, to overcome these barriers, MSF, MoH and patients piloted a community-based ART delivery model through “Community ART Groups” (CAGs), in which patients take an active role in ART provision in the community. Patients stable on ART form groups of up to six patients, taking turns to collect ART drugs for group members at the health facility. This rotation system reduces the frequency of clinic visits from 12 to a minimum of twice yearly. Each group elects a group leader, who functions as a spokesperson of the group. The group members meet regularly in the community, perform monthly pill counts and offer mutual adherence support. Lay counsellors, salaried by MSF, assist in forming and monitoring the groups in health facilities and the community [5]. Of the total 5782 adult members included in CAG between February 2008 and December 2012, 30% were male. A preliminary data analysis at the end of 2012 found a 95.7% retention rate after a median follow-up time of 19 months [6].

A qualitative evaluation of the CAG model revealed its dependency on external resources, questioning its future sustainability. This study highlights the components, which might facilitate and/or jeopardize the sustainability of the CAG model, and formulates recommendations to guarantee its long-term sustainability.

## Methods

### Qualitative study

We carried out a qualitative assessment of the CAG model's functioning, looking at group dynamics and the model's impacts on health outcomes, individual patients, health services and the broader community. Inductive qualitative content analysis was used to analyze the data collected through semi-structured interviews. Sixteen focus group discussions (FGDs) and 24 in-depth interviews (IDIs) were conducted among the five main stakeholders involved in the CAG model: (1) Patients on ART in groups and in individual care; (2) MoH Nurses; (3) MSF lay counsellors; (4) Health authorities; and (5) MSF implementers, a core team of MSF workers involved in the initial creation and implementation of the model (Table 1). All FGDs and IDIs were digitally audio-recorded, transcribed and coded, using NVivo 9 software (QSR International, Doncaster, Vic., Australia). After a coding verification process, the data were condensed and categorized into broader themes [7]. A more detailed description of the methods can be found elsewhere [8]. One of the emerging issues was the MSF dependency and the potential sustainability of the CAG model.

**Table 1:** Stakeholder groups interviewed in the focus group discussions and in-depth interviews

Stakeholder groups	Number of IDI	Number of FGD	Number of participants
1. Patients on ART^a^	15	12	79
In groups^b^	4	12	68
Returned to individual care	4		4
Remained in individual care	7		7
2. MoH nurses	1	2	10
3. MSF lay counsellors^c^		2	7
4. Health authorities (district, provincial and national)	5		6
5. MSF CAG implementer	3		3
Total	24	16	105

ART – anti-retroviral therapy; MoH – Ministry of Health; MSF – Médecins Sans Frontières; CAG – Community ART Groups.

a Patients on ART have been divided in three main groups: (1) patients in groups – CAG members and group leaders, (2) patients who returned to individual care after being in a group and (3) patients who preferred to remain in individual care.

b Fifty-one percent of the interviewed patients on ART in groups were male and 49% female.

c Counsellors are appointed to large health facilities, taking a major role in the daily management of the CAG activities. Whereas in smaller health facilities, nurses are responsible for these activities. During the interviews the nurses have been divided in two groups: (1) nurses working with counsellors and (2) nurses working without counsellors. Two of the seven lay counsellors interviewed were female.

### Ethical statement

Ethical approval to carry out the study was obtained from the ethical review boards of the Mozambican MoH and MSF. All the study participants gave written or verbal consent.

### Conceptual framework on the sustainability of community-based models

Sustainability of healthcare programmes can be defined as: “… the capacity to maintain programme services at a level that will provide ongoing prevention and treatment for a health problem after termination of major financial, managerial and technological assistance from an external donor” [9].

To identify the different factors which might influence the sustainability of community-based programmes, we searched the literature (PubMed, Google scholar and references of review articles) using a list of synonyms related to “programme sustainability” and “community-based models.”

**Figure 1:**
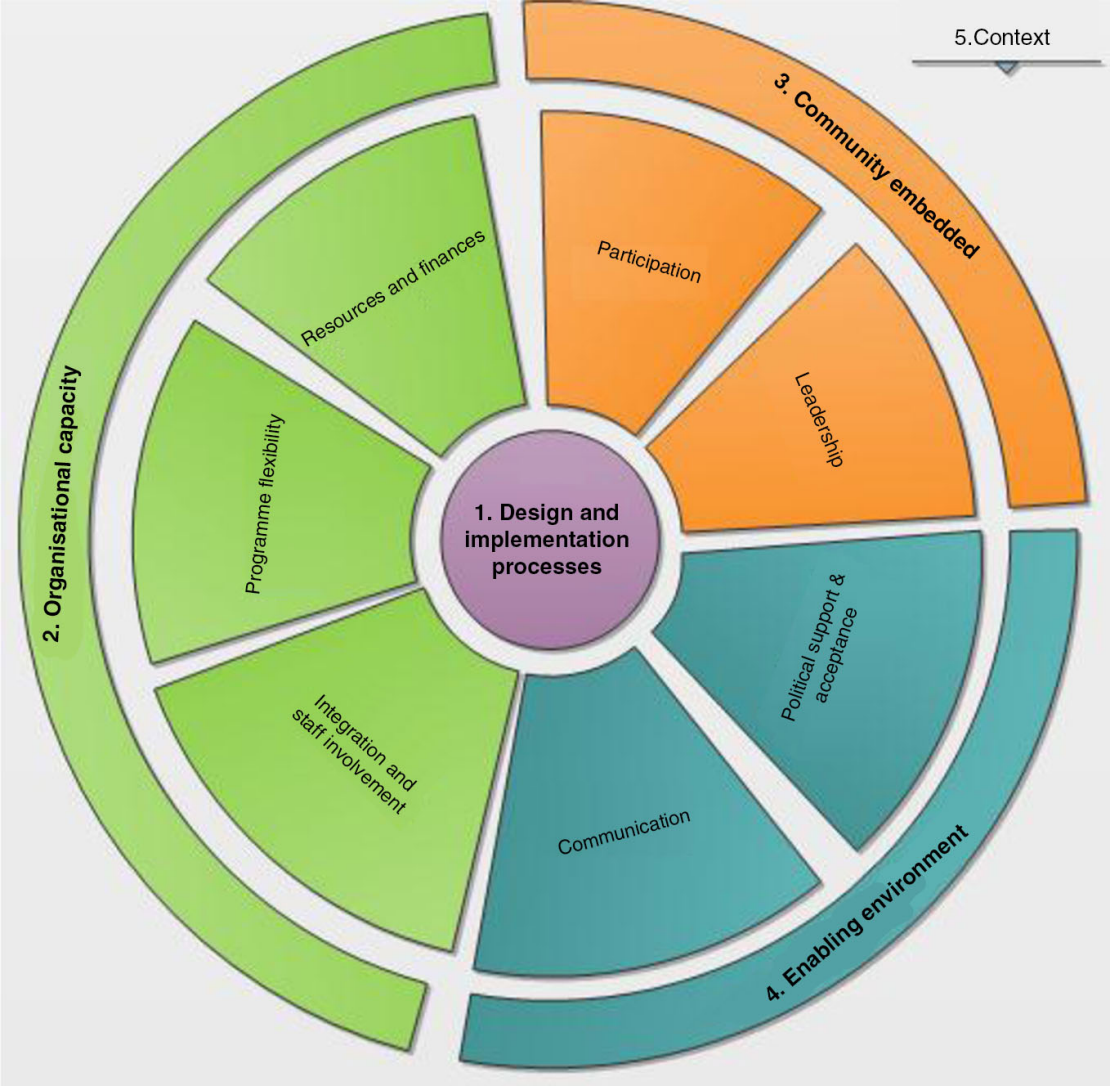
Conceptual framework on the sustainability of community-based models.

We developed a conceptual framework to explain the different components potentially influencing the sustainability of community-based programmes. This framework draws upon the work of Schell *et al*. [10] and Sarriot *et al*. [11]. It comprises five main components: (1) Design and implementation processes, (2) Organizational capacity, (3) Community embeddedness, (4) Enabling environment, and (5) Context (Figure 1).

*Design and implementation processes* (Figure 1: 1) refer to strategies and activities put in place to effectively reach the expected project objectives and goals, including the design of activities, negotiation processes, and training and capacity building [10–12]. *Organizational capacity* (Figure 1: 2) refers to the means and conditions required for the model to function independently, maintaining the core activities. This includes resources and finances, integration and staff involvement, and the programme's flexibility to adapt to beneficiaries’ needs [13]. *Community embeddedness* (Figure 1: 3) refers to community participation and leadership, encouraging PLHIV to take more responsibilities in the care of their chronic diseases. The fourth component, *enabling environment* (Figure 1: 4) includes political support and acceptance, the ability to efficiently communicate the project's outcomes and activities to obtain high visibility [10,14]. All these components are embedded in a broader *context* (Figure 1: 5) with political, economic, cultural and geographical factors, which are not directly controllable. The context needs to be considered when developing and implementing programmes in order to obtain long-term results [14,15].

## Results

We reported the broad spectrum of information retrieved during the interviews according to the five components of the conceptual framework on sustainability described above (Table 2). Sustainability, however, is a complex process, resulting in several dynamic interactions between these different components.

**Table 2:** Summary of the main factors potentially favouring or jeopardizing the sustainability of the Community ART Groups model according to the five components identified in the framework on sustainability

Components to sustainability	Favouring factors	Jeopardizing factors
	Based on patients’ reported needs – mainly barriers to access ART in the individual health services	
CAG model design and implementation processes	Stepwise implementation: consultation and negotiation processes with all stakeholders	Need for continuous supervision, training and capacity building
	Concept of self-management and patient empowerment to reach effective results	
Organizational capacity	Progressive MoH involvement and integration of activities in existing health services Flexibility to adapt to changing patients’ needs over time	Additional resources required in contrast to limited MoH resources available, especially the need of a “regulatory cadre” (e.g. counsellors) to form and monitor groups
Community embedded	Community participation – uniting people with common needs to take more responsibilities in the healthcare of their own and their peers	Some limitations emerged when shifting tasks and responsibilities to patients
	Leadership – patients are considered as partners in healthcare	
Enabling environment	CAG model is well accepted by all stakeholders	
	Changed mindset of all stakeholders concerning the new healthcare approach	Some barriers to access or join CAG remain
Context	Builds on social and cultural values and habits	Patients’ low basic knowledge and education level Poverty conditions Weak health system and poor healthcare coverage
	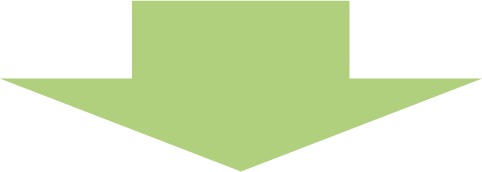	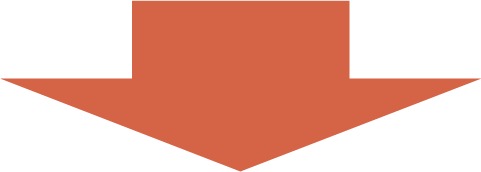
	These factors can be transformed in opportunities to reinforce the sustainability of the CAG model.	Some factors should be avoided to prevent them becoming threats. While some contextual factors will need to be addressed.

### 1. The CAG model design and implementation processes

To better understand the barriers to access ART and identify the needs, several consultation rounds were organized with patients (Textbox 1: Q1–3). Subsequently, to reach consensus, patients, health staff and health authorities met regularly during the stepwise implementation process. The model was progressively rolled out first in the rural areas and later in the more urban areas and adapted to the social context (Textbox 1: Q4).

**Textbox 1:** CAG model design and implementation

Based on patients’ reported needs – mainly barriers to access ART care in the individual health services	*Q1 – Most patients do not have the means to go every month to the hospital, to obtain money to go every month. Lots of people died, only the ones living close to the hospital survived. –* IDI with CAG member from rural area *Q2 – People were feeling bad, they thought the problem I have is only my problem without knowing that this problem was affecting the majority, lots of people*. – IDI with Group leader from rural area *Q3 – …the needs and motivation to join groups are very different. In town, the most common complaint is the long waiting time in the health facilities. […], in the contrast to rural areas, in town problems related to distance and transport do not exist …* – IDI with MSF implementer
Stepwise implementation: consultation and negotiation process with all stakeholders	*Q4 – I think there were consultations in the sense that when we started to form CAGs, we listened to what people wanted, what their needs were to be able to adapt the activities accordingly […] it is not by imposing … a solution. You cannot do this […] you have to take into account the cultural problems. It is only by being in the community you will perceive these problems and understand the day-to-day needs of the patients. –* IDI with National health authority
Concept of self-management and patient empowerment to reach effective results	*Q5 – The responsibilities of the healthcare worker decreased with the CAG model in which patients take a more active role in the care delivery, they are no longer patients, this is perceived as a big help for the healthcare workers*. – IDI with District health authority *Q6 – In the past our situation was very bad, now we recovered, we are in a good physical condition and are happy. Also we live well and are able to work … –* CAG member during FGD with CAG members from rural areas *Q7 – The discrimination seems to have finished because lately the people in the community lost track of who is receiving drugs as most of us are in good physical condition. They stopped to think that we are ill because before we went regularly to the health facilities, but today we are not going anymore … –* CAG member during FGD with CAG members from rural areas
Need for continuous supervision, training and capacity building	*Q8 – We need more training related to monitoring and auto-monitoring of the groups, because these are technical aspects which need a better knowledge … –* IDI with District health authority *Q9 – … as we mentioned, we need capacity building for group leaders … to be able to continue these activities in the future. –* Group leader during FGD with group leaders from semi-urban areas

The concept of the CAG model is based on the principles of self-management, providing more responsibilities to the patients for their own healthcare (Textbox 1: Q5). This approach resulted in an effective ART delivery model with direct benefits for the patients and the health services, and extended impacts on the broader community. All participants agreed that benefits for the patients – mainly cost and time savings, mutual adherence support and increased assurance of timely access to ART, timely – together with benefits for the health facilities – decreased workload and better monitoring of patients – resulted in better general well-being, less LFU and deaths, and improved adherence to treatment (Textbox 1: Q6). In addition, several indirect impacts were identified such as an increased HIV awareness, an improved health-seeking behaviour, including an increased uptake of HIV testing, and a reduction of stigma (Textbox 1: Q7).

However, continuous supervision, training and coaching sessions for patients and health staff was thought to be essential to ensure the well-functioning of the groups and the quality of care (Textbox 1: Q8–9).

### 2. Organizational capacity

Despite the increased MoH involvement over time, the daily management of the CAG model still depends strongly on MSF support (Textbox 2: Q1). An “additional cadre” is needed to regulate the process, to link patients into groups, and to coach and to monitor the CAGs (Textbox 2: Q2–3). Some counsellors suggested paying health staff incentives to motivate them, while a district health authority mentioned the need to consider incentives to reward group leaders for their work and permanent availability (Textbox 2: Q4). Besides human resource requirements, healthcare workers considered the lack of transport a major obstacle to organizing regular supervision visits in the community. These addtional resources required are in sharp contrast to the current resources and capacity of the MoH (Textbox 2: Q5).

**Textbox 2:** Organizational capacity

Progressive involvement of MoH staff and integration of activities in existing health services	*Q1 – Often the groups are considered as a MSF project with little involvement of the MoH, however this improved a lot lately, MoH staff are much more involved in the care and the groups are considered as the work of everybody*. – Counsellor during FGD with counsellors
Additional resources required in contrast to the limited MoH resources available, especially the need of a “regulatory cadre” (counsellor) to form and monitor groups	*Q2 – The counsellor organizes the groups because no one in the community who knows better our situation, our problems, … only the counsellor does. Of each area, the counsellor knows exactly how many people are on ART and how many groups there are. Of each area! (S)he is our maximum chief, who has all the information. –* Group leader during FGD with group leaders from rural areas *Q3 – The counsellor is essential for the group to function, without the counsellor the groups will not continue as he is the only one who knows the people in the community*. – Nurse during FGD with nurses working with counsellors *Q4 – … need to incentivise patients for their active role in the health facilities for example through extra trainings to keep them motivated …* – IDI with District health authority *Q5 – … this programme is monitored by MSF, which has resources, and means, I do not have …, I notice how … how this functions. OK! A team comes, goes to the communities, organizes the groups … […] but I wonder […] Do the districts have these resources and capacity to continue these activities? –* IDI with District health authority
Flexibility to adapt to changing patient needs over time	*Q6 – A period of six months before being able to join CAG, cannot be, because is too long … because for us to evaluate if a person is adherent to treatment or not, two months should be sufficient to judge if a patient is adherent and stable or not and can join CAG*. – Nurse during FGD with nurses working with counsellors *Q7 – There is a need to adapt the entry criteria according to the needs of the patients and the context, the criteria need to be flexible for changes, […] new target groups might benefit for the CAG model, for example TB patients, pregnant women, patients on second line treatment …* – IDI with MSF implementer

Moreover, to remain functional and beneficial, the CAG model has to be flexible, continuously adapting to patients' needs. Many stakeholders mentioned a broad application of medical entry criteria, allowing patients with less than six months on treatment and/or a lower CD4 count to join CAG or at least participate in the group dynamics (Textbox 2: Q6). Also, other more vulnerable target groups (children, pregnant women, patients with TB, those not eligible for ART, on second line ART or other chronic diseases) could benefit from the peer support generated by the CAG model (Textbox 2: Q7).

### 3. Community embeddedness

The CAG unites people in a similar situation. The possibility to share common needs and problems breaks the patients’ isolation and reinforces their motivation to adhere to treatment. It was often mentioned that patients develop a strong bond being in CAG, considering themselves as a new family and/or church (Textbox 3: Q1).

**Textbox 3:** Community embeddedness

Community participation – uniting people with common needs to take more responsibilities in the healthcare of their own and their peers	*Q1 – Now that we are taught to be together, united, we care for one another, we became one head, one body and one heart, because everything we do is the same. –* CAG member during FGD with CAG member from rural areas *Q2 – We have to share with the patients their needs and their desires, listen more to them, leave more openness for CAG members to participate in their day-to-day healthcare, […] they are considered as partners of the national health system […] leave the patients to talk for themselves and take decision in relation to their day-to-day lives, their health, they have to participate and have to have the space to participate …* – IDI with MSF implementer
Leadership – patients are considered as partners in healthcare	*Q3 – … everything changed, I'm well known in the area, because I'm in charge of patients, whenever a person is sick, can be a child, mother, father, they come to my house, they are told to come to my house, when I see that this illness has nothing to do with ours I accompany them to the hospital*. – Group leader during FGD with group leaders from semi-urban areas *Q4 – The community has to take an active role in the fight to prevent diseases in the community … they could form committees or associations in the community to monitor and active participate in the health related issues for prevention of STI, malaria, etc … –* IDI with Provincial health authority
Some alarming voices on the limitations of task shifting	*Q5 – … members cannot be turned into mini-nurses overnight*. – IDI with District health authority

Moreover, CAG members are considered partners in health (Textbox 3: Q2). In some health facilities, patients actively participate: packing drugs, arranging patient files, counselling patients, checking pill counts and tracing defaulters. Some function as an information feedback loop between the health facilities and the communities: giving health talks, sensitizing people for HIV testing, identifying and accompanying sick people to the health facilities. Others are even regularly approached for general medical advice by people in the community (Textbox 3: Q3).

Some participants proposed providing CAG members more responsibilities through associations or committees: participating in prevention activities (family planning, vaccination campaign, nutrition activities, etc.), performing HIV testing or managing first aid kits and/or stocks of ART drugs in the community (Textbox 3: Q4). Also, income-generating projects were generally welcomed to cover the costs of the groups and were thought to be able to attract more people to join CAG.

Despite the encouraging voices to support patient empowerment, district health authorities warned about the possible harm created when employing lay people in the health system (Textbox 3: Q5). For example, some patients in groups reported incorrect beliefs about HIV and ART, such as the need to interrupt ART when on TB treatment, the risk of transmitting HIV using food utensils or blood group O being protective against HIV.

### 4. Enabling environment

Generally, the CAG model was well accepted (Textbox 4: Q1). Initially, many health staff and health authorities were rather sceptical about the changes proposed. Over time, being confronted with positive health outcomes and the additional benefits of the CAG model, they became more enthusiastic and considered the model a good alternative option to retain patients in ART care (Textbox 4: Q2).

**Textbox 4:** Enabling environment

CAG model is well accepted by all stakeholders	*Q1 – I can say that the groups are very welcome, the patients are very satisfied with the formation of the groups, I want to say that we have to continue, […] because in the future we will have more patients and at least the groups help us. The groups help us a lot … –* IDI with nurse working without counsellors *Q2 – Practice showed that CAG is the best alternative model to overcome the barriers of individual care*. – IDI with District health authority
Changed mindset of all stakeholders concerning the new healthcare approach	*Q3 – We have to have the confidence that patients can be responsible and can take care of their health …* – IDI with National health authority *Q4 – We cannot stop to sensitize for a mentality change, a change in approach, a change in perception of the proper CAG strategy. It is a new strategy for the MoH, we can say it is very new. That's why we have to work hard to change the perception regarding the CAG dynamic*. – IDI with MSF implementer
Some barriers to access or join CAG remain	*Q5 – Myself, I think that remaining two three months without going to the hospital, brings along some problems for the patient*. – IDI with patient who remained in individual care *Q6 – People are afraid to join CAG as others will discover that they are infected and no one will want to marry them*. – Group leader during FGD with group leaders from remote areas

Hence, the CAG model requires a change of mindset of all involved partners (Textbox 4: Q3). Participants highlighted the continuous need to sensitize and inform people of the advantages of the CAG model (Textbox 4: Q4).

Nevertheless, some barriers to joining CAGs still remained. Several patients were not aware of the CAG model due to poor information flow and coordination. Others opted not to join due to certain perceptions. For example, they doubted the quality of care offered through the CAG model or were afraid of the consequences of joining a CAG (Textbox 4: Q5).

### 5. Context

The CAG model builds on cultural and social habits and values, creating strong social links and networks between members (Textbox 5: Q1).

**Textbox 5:** Context

Builds on social and cultural values and habits	*Q1 – The social affinity between people in our culture demonstrates the social connection that we have, which is very compatible with the principles the model is based on*. – IDI with MSF implementer
Patients’ low basic knowledge and education level and poverty conditions	*Q2 – … we can even say that several members are illiterate; so they have to be taught. In a simple way, but they have to be taught, taught and remembered constantly …* – IDI with district health authority *Q3* – *Some patients sold chickens to at least obtain money to pay transport to collect drugs, sold goats …* – Member during FGD with CAG members from rural areas
Weak health system and poor healthcare coverage	*Q4* – *We know that the district is very large … for example, here in the district X we only have three health facilities which provide ART care, they do not cover the entire population of the district. Ideally, every health facility should have health staff trained and capacitated to respond to the demands. The people are located in small concentration at very large distances, without possibility to access treatment, because of these large distances and the lack of transport means to the health facilities …* – IDI with District health authority *Q5 – The CAG model is an initiative which involves going to the community, but it only exists where the health facilities already cover the needs […] the areas supported by the CAG model are already covered by the national health services, do you understand? We already cover these areas. […], but there is 40% of the population we are currently not able to cover. –* IDI with National health authority

District health authorities called for caution as the basic knowledge and education level of most patients is limited (Textbox 5: Q2). Also, the extreme poverty conditions limit the capacity of the patients to travel and access care, especially people living in remote areas (Textbox 5: Q3).

The current national health system deals with a chronic shortage of health staff and poor healthcare (Textbox 5: Q4). Likewise, national health authorities found that the CAG model has a limited impact on the overall access to ART as the main focus of the CAG model is on retention on ART (Textbox 5: Q5).

## Discussion

Sustainability of programmes is essential to roll-out healthcare interventions and maintain improved health outcomes [16]. This qualitative analysis based on a conceptual framework on sustainability of community-based programmes demonstrates several arguments favouring or arguing against the sustainability of the CAG model.

**Figure 2:**
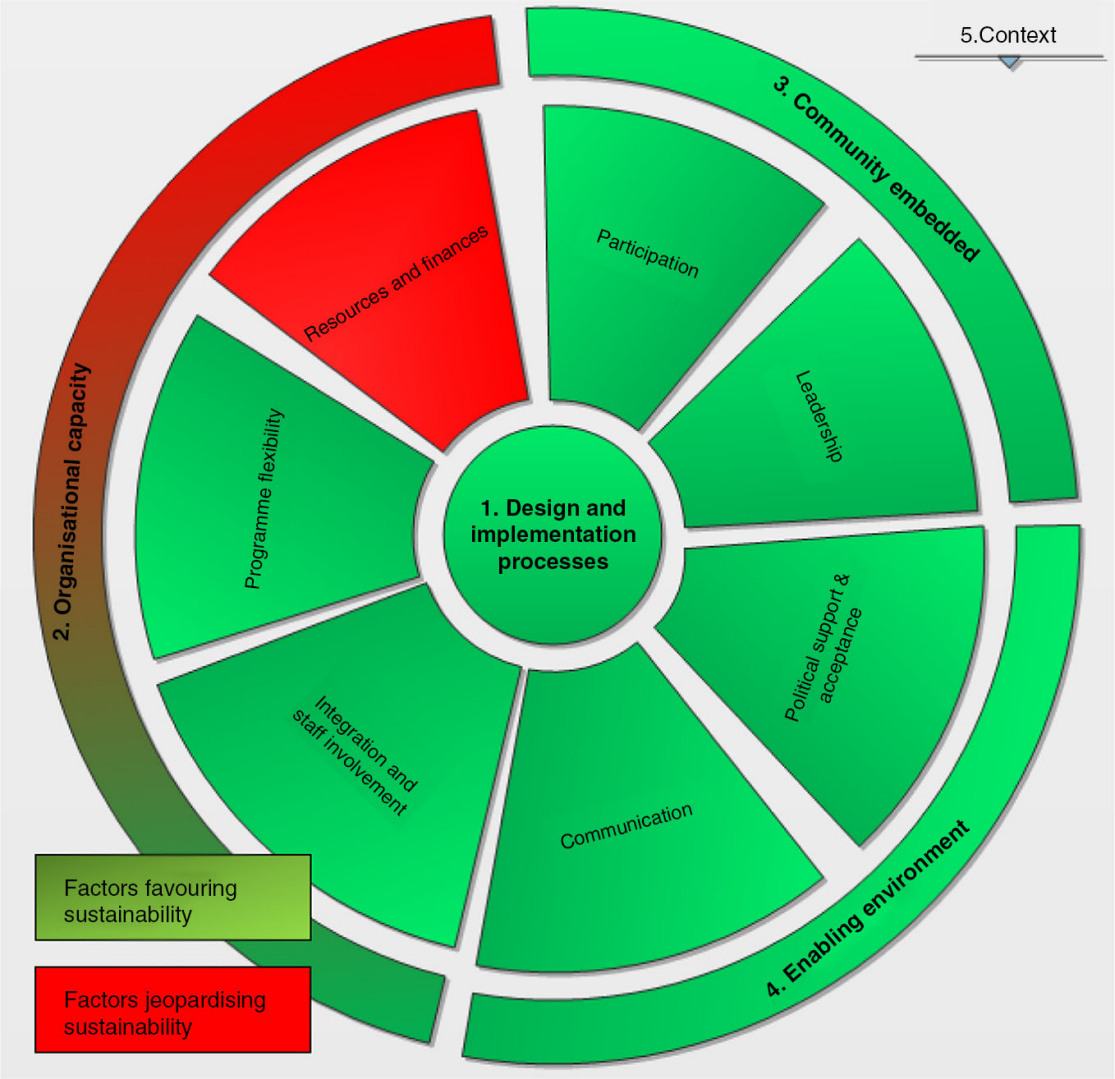
Strong and weak components of sustainability CAG model – based on conceptual framework.

The major arguments in favour of the sustainability of the CAG model are the effectiveness of the model, the flexibility to adapt to the patients’ needs, community embeddedness and ownership, and the high acceptance of the model. One of the main factors jeopardizing the model's sustainability is the high dependency on MSF resources (human, financial and logistical) to ensure good daily functioning of the model (Figure 2).

Programmes proven to effectively reach their goals and targets tend to be more sustainable [14]. All participants agreed that through the CAG model health outcomes, adherence and retention in care improved significantly. These positive outcomes can only be obtained and sustained by involving patients and community in healthcare. According to Rifkin *et al*. community participation can be considered through two different approaches: (1) a *target-oriented, top-down approach* whereby activities are defined and decided upon by the healthcare providers, involving the community as a “passive” means to reach the objectives of the programme; (2) a *bottom-up approach* focusses more on *empowerment of the community*, providing the community an “active” voice in the decision-making process, implementation and planning of activities [17]. Evidence shows that the first approach has not been very successful in the past, whereas the second is not only more effective but also more acceptable and accessible as it is often based on trust and respect [16,18,19].

When constructing the community participation from a bottom-up approach, it is key to consider the beneficiaries’ perceptions of their own health, motivation and needs, as often health problems are not the patients’ main concerns. Therefore, an essential first step is to create a platform through community gatherings, where PLHIV can voice their needs. This platform can later serve as an information feedback loop to adapt activities according to changing needs [20]. This implies a dynamic process, in which people progressively gain more control over their health situations and health outcomes [18,21]. Prior to the implementation of the CAG model, the patient–doctor relationship reflected a hierarchical control over patients, who played a passive role [22]. The CAG model created a new way of thinking in healthcare provision, recognizing and respecting patients as partners in healthcare [18,23,24]. The analysis highlights the emerging active role patients perform in health facilities and communities, taking progressively more responsibilities in drug distribution, counselling and surveillance activities.

In addition, Rifkin *et al*. highlighted four essential elements needed to reinforce community participation: (1) a common geographical area, (2) common interests, (3) feelings of ownership and (4) social relationships [18]. Likewise, the CAG model unites PLHIV from the same geographical area, all living with the same difficulties and challenges in accessing ART. It stimulates a sense of ownership and reinforces existing social networks.

Moreover, the extent to which the model is accepted and implemented in different health facilities is a good indicator of the success of the innovation and the potential future sustainability [25]. The acceptance process of the CAG model is comparable with the acceptance curve of innovative programmes and products described in the marketing sector, with relatively strong initial resistance from different levels as people needed to gain knowledge and confidence in the model [26,27]. Endorsement increased steadily once the larger benefits and the impact of the CAG model became obvious. Nevertheless some health authorities and healthcare workers not well acquainted with the model still show some level of resistance and are less likely to adopt the model.

Despite these arguments favouring the sustainability of the CAG model, we have to be cautious for other factors which might jeopardize its sustainability. Several participants highlighted the persistent dependency of the model on MSF, with counsellors and MSF implementers often called as a “fire brigade” to solve problems in groups.

In spite of the “*bottom-up approach”* of the CAG model, it became obvious that an “additional cadre” is required to regulate the process, to broker patients’ confidentiality, to link them into groups, to monitor the group dynamics and to intervene when problems occur. Also, the continuous need for training and supervision to maintain quality of care should not be overlooked [24,28]. To date, counsellors employed by MSF have mainly been in charge of these functions. However, MSF will scale-down its support over the coming years. So the question remains who will embrace these responsibilities in the future: counsellors, nurses or patients themselves? Despite ongoing lobbying and advocacy activities, the cadre of lay counsellors has not yet been officially recognized in Mozambique. Nurses already have a broad variety of responsibilities, often not allowing them to dedicate sufficient time to the daily monitoring and management of the groups. Another option is to increase the patients’ responsibilities in CAG monitoring and management. However, bearing sustainability in mind, it is important to not consider patients as a cheap solution to bypass the weaknesses of the health system [22]. A balance between the patients’ investment versus the benefits they receive has to be safeguarded, in order not to jeopardize the volunteerism principle on which the CAG model is based. When providing more responsibilities to the CAG members, remuneration may need to be considered in order to retain them in the programme, and to hold them accountable for their activities, such as HIV testing [29,30]. Health authorities have already highlighted the need to provide incentives to CAG members. Future follow-up will be required to monitor long-term motivation, as communities might suffer from participation fatigue over time, requiring new roles and challenges to maintain their motivation and support [31].

Moreover, sufficient means (transport, incentives, etc.) need to be secured to supervise the groups and their dynamics. To maintain good quality in CAG activities, the overall health system will need strengthening, ensuring adequately trained health staff, uninterrupted drug supply and logistical means to perform supervision activities.

Finally, the CAG model should be considered as one possible approach to overcome the access barriers. A choice should be offered to patients as to which approach suits best their needs and preferences. There is no one-size-fits-all solution. Despite the initial successes, a “CAG-like approach,” currently piloted on a large scale in Mozambique, will need to be adapted to the local context, needs and resources available [18]. Moreover, further research is required to evaluate the suitability of the model for other target groups, for example, TB patients, children, pregnant women and patients on second line ART.

The strengths of the study are the large number of stakeholders interviewed and the verification process during data collection, transcription and translation to assure quality of the data. The major limitations are first, the possible selection and recall bias of participants interviewed. Second, as priority was given to the language skills of the local research team compared to their prior experience in qualitative research, we sometimes had to compromise on some steps of the qualitative research and iterative reflection process. Third, as the conceptual framework was developed after data collection not all components were equally raised during the in-depth interviews with key informants.

## Conclusions

Based on our conceptual framework (Figure 2), we identified strong components favouring the sustainability of the CAG model, the strongest being community participation and leadership. Furthermore, the growing enabling environment with increasing political will, and progressive MoH involvement in the daily management of the CAG model, will facilitate its future sustainability.

Nonetheless, the weak link in the chain remains the resources required to maintain CAG activities. The major obstacles are (1) the need of human resources: the essential “regulatory role” to link patients and monitor the groups, as well as (2) the logistical constraints to supervise and support the CAG model. Both could benefit from strengthening of the overall health system.

Despite strong community participation in the CAG model, the new responsibilities taken up by PLHIV mainly focus on the implementation of the daily activities within the groups. In the future, it will be important to reinforce community participation by involving the community in the planning process of the model, while reflecting on possible solutions for the “regulatory” function required.
